# Predicting high health-cost users among people with cardiovascular disease using machine learning and nationwide linked social administrative datasets

**DOI:** 10.1186/s13561-023-00422-1

**Published:** 2023-02-04

**Authors:** Nhung Nghiem, June Atkinson, Binh P. Nguyen, An Tran-Duy, Nick Wilson

**Affiliations:** 1 Department of Public Health, University of Otago, Wellington, New Zealand; 2 School of Mathematics and Statistics, Victoria University of Wellington, Wellington, New Zealand; 3 Centre for Health Policy, Melbourne School of Population and Global Health, University of Melbourne, Melbourne, Australia

**Keywords:** Machine learning, High-cost users, CVD cost prediction, Health and social administrative data, New Zealand, C55, I15, N37

## Abstract

**Objectives:**

To optimise planning of public health services, the impact of high-cost users needs to be considered. However, most of the existing statistical models for costs do not include many clinical and social variables from administrative data that are associated with elevated health care resource use, and are increasingly available. This study aimed to use machine learning approaches and big data to predict high-cost users among people with cardiovascular disease (CVD).

**Methods:**

We used nationally representative linked datasets in New Zealand to predict CVD prevalent cases with the most expensive cost belonging to the top quintiles by cost. We compared the performance of four popular machine learning models (L1-regularised logistic regression, classification trees, k-nearest neighbourhood (KNN) and random forest) with the traditional regression models.

**Results:**

The machine learning models had far better accuracy in predicting high health-cost users compared with the logistic models. The harmony score F1 (combining sensitivity and positive predictive value) of the machine learning models ranged from 30.6% to 41.2% (compared with 8.6–9.1% for the logistic models). Previous health costs, income, age, chronic health conditions, deprivation, and receiving a social security benefit were among the most important predictors of the CVD high-cost users.

**Conclusions:**

This study provides additional evidence that machine learning can be used as a tool together with big data in health economics for identification of new risk factors and prediction of high-cost users with CVD. As such, machine learning may potentially assist with health services planning and preventive measures to improve population health while potentially saving healthcare costs.

**Supplementary Information:**

The online version contains supplementary material available at 10.1186/s13561-023-00422-1.

## Introduction

It is widely acknowledged that healthcare cost distributions are typically highly skewed to the right, indicating that a small proportion of patients consume a disproportionate amount of health care resources. For example, the highest-cost decile of patients (*n* = 1486 out of 14,855) accounted for 60% of total hospital and primary care services costs in a US study [1]. In another study, 13% of patients consumed up to 87% of healthcare in various US hospital settings [2]. High-cost users also accounted for long hospital stays and other health resources in Australia [3].

There is substantial heterogeneity within the high-cost patients, determined by a wide range of health events and social economic conditions. For example, one study reported that patients with a diagnosis of subarachnoid haemorrhage, acute respiratory failure, or complications of surgery procedures, were more likely to incur high cost [4]. Another study reported that repeated hospitalisations for the same disease were more a characteristic of the expensive patients than were single cost-intensive stays, or prolonged single hospitalisations [2]. Long-term conditions such as heart failure, [5] end-stage renal disease, angina, and depression are a key characteristic of high-cost users [3]. Persistent high-cost users were more likely to concurrently use multiple chronic medications [6]. Ageing of the population is also associated with an increase in the proportion of high-cost users of inpatient care [3]. Similarly, patients who live in poorer neighbourhoods, and have addiction issues have been reported to be more likely to be in the high-cost user group [2, 7–11]. Given these heterogeneities within high-cost user groups, there is a need to look at specific diseases and to design specific interventions in order to improve population health [6] and to better plan for health services demand.

Cardiovascular disease (CVD) is expensive to treat and is a leading economic burden in terms of costs to health systems [12–14]. Furthermore, it is the leading cause of death and morbidity in many countries, with an estimated of 17.8 million deaths worldwide annually [15]. This disease burden continues to rise in almost all low- and middle-income countries, and even has begun to rise in some high-income jurisdictions where it was previously declining [16].

The standard practice in predicting CVD and other health cost distributions is to employ a regression model with well-established risk factors, such as age, sex, ethnicity, education and socio-economic position [12, 17]. This approach could be useful for policy interventions at a population level, but is likely to perform poorly at a group- or individual-level, as it does not capture many aspects of patient heterogeneity which are of importance for designing the interventions [6]. In addition, high-cost users are in the minority in healthcare settings, that is a small number of users typically account for a large proportion of the total healthcare costs [2]. Therefore, using traditional regression methods to predict high-cost users could lead to unstable estimates. Relationships and interactions among variables can be non-linear and complex, and it is impractical to diagnose and include all functional forms and interactions in traditional regression models [18, 19]. Furthermore, as rich health and social administrative data become increasingly available, the traditional regression methods are not ideal to handle big datasets efficiently [18, 20].

Data science and – in particular – machine learning, offers a potential improved and more accurate risk prediction, and efficiently utilises available information in an era of big data (i.e., large quantities of data [11]) [21, 22]. In the economics field, machine learning methods are increasingly recognised as an important tool for data analysis. In particular, “*Machine learning not only provides new tools, it solves a different problem. Specifically, machine learning revolves around the problem of prediction, while many economic applications revolve around parameter estimation. The success of machine learning at intelligence tasks is largely due to its ability to discover complex structure that was not specified in advance*” [18, 20]. While machine learning methods have been increasingly employed in recent years in the prediction of health outcomes [19, 23, 24], its use in health economics is still limited, with only a small number of studies with regard to CVD being published [25, 26].

This study aimed to predict high-cost users among people with CVD using machine learning methods and rich administrative data in a high-income country (New Zealand). Specific objectives are: (1) to use a new tool on available rich individual-level data to better address heterogeneity issues, and (2) to identify potential new risk factors of high-cost utilisation.

## Methods

We used data from New Zealand as it has national linked administrative datasets. We focused on people with CVD because CVD costs NZ$3.3 billion annually, makes up around 14% of all health loss nationally, is responsible for a third of the total number of deaths annually and is ranked as the top cause of deaths in 2019 [27–29]. The national datasets include health-related variables (e.g., hospitalisation, pharmaceutical prescriptions, laboratory tests requested, health conditions) and sociodemographic data (e.g., country of birth, educational level, income, deprivation index [30]). The model outcome (i.e., the dependent variable) was the most expensive cases, i.e., those in the top 20% of the people with CVD who had highest health care costs over a one-year period in 2014. We used four machine learning models to predict health costs, and compared their performance with traditional regression models (logistic regression). In the sensitivity analysis of the impact of the high cost threshold on the model performance, we ran and compared the machine learning and traditional models when the thresholds for high cost was set at 5%, 10% and 30%.

### Description of datasets

The Stats NZ Integrated Data Infrastructure (Stats NZ IDI) is an integrated data environment that links longitudinal microdata about individuals, households, businesses and organisations [31]. All individual data were linked through the Central Linking Concordance (the Spine) – the backbone of the integrated data which aims to identify all individuals who have been resident in New Zealand. The Spine links to each of the main datasets used in this study. Aggregated tables use multiple data sources to provide basic demographic information for each individual, including sex, month and year of birth, and ethnicity indicators.

There were four main datasets used in this study: the first dataset was Census 2013 to identify individuals’ smoking status, language spoken, employment status and other demographic information (see Table 1 for more details). The Census 2013 dataset is the count of people and dwellings enumerated by the population census on census night (5 March 2013). The second dataset, the CVD dataset from the Ministry of Health (MoH chronic condition table), contains information about healthcare users in the whole New Zealand residents. We used this dataset to identify people with CVD and other chronic diseases, [32] which was available from 1/1/2002 to 31/12/2015 [33]. We also used pharmaceutical data from 2013 to identify individuals on CVD preventive pharmacotherapy (e.g., statins). The Pharmaceutical Collection contains claim and payment information from pharmacists for subsidised dispensings. Finally, we used the IDI Population Explorer dataset (2013), which summarises information about individuals available within the IDI, with indicators in 2013 for receipt of social security benefit, use of social housing, major life events (i.e., getting divorced/separated when this was officially documented); and a criminal record [34].

**Table 1:** Description of socio-demographic and health variables used in the modelling – general population and people with CVD (aged 30–74 in 2013)

Variables	General population		People with CVD	
	N	Percentage (%)	N	Percentage (%)
**Total study population**	1,889,000	100%	97,950	100%
**Ethnicity**:				
**Māori** (Indigenous New Zealanders)	208,000	11%	11,754	12%
**Non-Māori**	1,670,000	89%	85,217	87%
**Country of birth:** Oceania (including New Zealand, Australia and Pacific)	1,450,000	77%	80,319	82%
**Age:** mean (in years)	49.7		62.7	
**Sex**				
Male	882,000	47%	64,647	66%
Female	1,007,000	53%	33,303	34%
**In paid employment**	1,341,000	71%	44,078	45%
**Social family status:** Spouse/partner	1,377,000	73%	68,565	70%
**Tobacco smoking status**				
Never smoked	1,064,000	56%	42,119	43%
Ex-smoker	478,000	25%	38,201	39%
Current smoker	275,000	15%	13,713	14%
**Official spoken language:**				
English	1,825,000	97%	94,032	96%
Te reo Māori (Māori language)	60,070	3.2%	3,918	4%
**Education (from Census):** Have a post-graduate qualification	839,000	44%	32,324	33%
**Potentially stressful life events** Divorced, separated, or widowed (as declared in the Census 2013)	233,000	12%	18,611	19%
**On a social security benefit in 2013** (= 1/0) (where the total of all types of benefits was greater than NZ$1000 annually)	207,000	11%	12,734	13%
**Residential property:** Own it (as opposed to rental)	1,205,000	64%	65,627	67%
**Individual income level** (stratification based on tax rates)				
Low-income (< NZ$30,000/year (US$20,750, €15,690))	681,000	36%	57,791	59%
Medium-income (> = NZ$30,000/year & < = NZ$70,000/year (US$48,400, €36,600))	669,000	35%	21,549	22%
High-income (> NZ$70,000/year)	384,000	20%	10,775	11%
**CVD** prevalence 2013	97,950	5.2%	97,950	100%
**Diabetes** prevalence 2013	149,000	7.9%	28,406	29%
**Other chronic conditions** namely chronic obstructive pulmonary disease, various cancers and traumatic brain injuries (further information see data dictionary [35])	272,000	14%	78,360	80%
**Use of CVD preventive medications in 2013** (at least 2 prescriptions)				
Any anti-hypertensive medication	368,000	20%	33,303	82%
Any lipid-lowering medication	245,000	13%	75,422	77%
Any prescribed anti-thrombotic agent	149,900	7.9%	75,422	77%
Any diabetes medication	88,600	4.7%	18,611	19%
**A history of atrial fibrillation (AF)**	27,000	1.4%	10,775	11%
**A history of any hospital event for hypertension**	2,820	0.2%	980	1%

Notes: Some numbers were not consistent due to random rounding and decimal places to comply with the IDI confidentiality rules

### Data linkage

For each individual, health and social characteristics from other datasets were added to the Census 2013 records using a unique SNZ ID, as long as they were in the IDI estimated New Zealand residential population in 2013. That is, data were not included for people who were out of the country for more than six months in 2013 or did not fill in the census. All the data processing steps were done using structured query language (SQL). See Figure S1 in the Appendix for data linkage visualisation.

### Study population

Only people who were in both the Census 2013 and the IDI estimated residential population in 2013 were included in the study. We also further restricted the population to people aged between 30 to 74 years old at the time of the census (in 2013). We selected this age range because of the relative rarity of CVD cases prior to 30 years of age and the presence of multiple comorbid conditions in people aged 75 years and older which were not fully captured in the Census and IDI [36]. Observations with missing age and sex information were very few and therefore excluded from the analysis. As a result, the total population used in this analysis consisted of 1,889,000 people (and the study population was further restricted to people with CVD). We used the definition of CVD from the Burden of Disease study in New Zealand based on ICD-10 codes to extract the study population (i.e., I60-I64 and G45-G46 for stroke, and I20-I25 for coronary heart disease) [7, 37]. Some expensive heart conditions were attributed to infant conditions and birth defects, such as congenital heart defects, or were redistributed to other conditions such as heart failure; hence these conditions were not included in our study. According to the NZ Burden of Disease study, [27] heart failure is a “sequela” (consequence) of several conditions, ranging from congenital cardiac malformation to coronary heart disease. Further information about disease definitions, including ICD-10 codes is provided in the Appendix Table S1.

### Outcome variables

The dependent variable was a binary outcome that indicates if an individual is a high-cost user of the health system costs in 2014 among people with CVD in New Zealand. In the base case, high-cost users were defined as people in the top 20% of those in the study population. In the sensitivity analysis, the threshold was changed to 30%, 10% and 5% to compare the prediction accuracy of machine learning methods with the traditional regression models.

### Explanatory variables

The unique Stats NZ IDI linked dataset allowed us to examine the effects of not only the health indicators (e.g., smoking status, diabetes), but also individual’s other demographic (age, sex, ethnicity) and social background variables, such as socio-economic position (deprivation [an area-based measure of socioeconomic deprivation] [30], income, education), potential stress indicators (via employment or family status), immigration, and social security benefits. A list of the variables was provided the Appendix Table S1.

### Machine learning and traditional models

We used four machine learning models that are common in the literature (i.e., L1-regularised logistic regression, classification trees, k-nearest neighbourhood (KNN) and random forest [18, 38–41]) to predict if an individual was a high-cost user. L1-regularised logistic regression is a penalised regression method using the sum of the absolute vector values for regularisation. L1-regularised logistic regression has an ability to reduce overfitting and shrink coefficients of unimportant variables to zeros, and hence, eliminate the need of applying feature selection on high dimensional data [42, 43]. A classification tree is a flowchart-like structure where each node represents a simple decision rule on a predictor variable that best separates the outcome into two groups with the most disparate probabilities of event. KNN is a non-parametric method of pattern recognition for classification or regression that makes decisions based on its neighbours, which are defined based on the distance between an observation and all other observations, for example the Euclidean distance; the k observations with the smallest distance are the k nearest neighbours. Random forest is an ensemble of decision trees created by using bootstrap samples of the training data and random feature selection in tree induction. Since this split is binary, it is able to capture non-linearities in the data, as multiple splits on the same predictor can occur within one tree. We selected these methods as they are commonly used in the literature, work with binary data, and have potential for interpretability, which is of interest to policy decision makers. Extensive discussion on pros and cons of these methods in health literature has been provided elsewhere [38]. A number of R packages were used to build the models: “LiblineaR” for L1-regularised logistic regression, “class” for KNN, “rpart” for classification trees, and “randomForest” for random forest. Tuning models’ parameters were selected with cross-validation, with some parameters being manually set based on model performances (i.e., number of trees in random forest, and k in KNN).

The traditional regression models were logistic regression models, which predict the probability of an individual being a high-cost user. In these models, we included only well-established CVD covariates available in the IDI: age, sex, ethnicity, smoking status, diabetes status, and other long-term conditions [17, 29, 44]. We also tested the models’ prediction accuracy (see section Model evaluation) with different sets of risk factors (i.e., excluding smoking status and ethnicity). We used R (version 3.3.0) for data compilation and model development [45].

In the sensitivity analysis of the impact of inclusion of variables on model performance, we ran: (1) the traditional regression models with a full set of all variables as those included in the machine learning models; and (2) the machine learning models with a limited set of variables as those included in the traditional regression models. Because the inclusion of ethnicity in the model may result in a stigma against particular ethnic groups in terms of excessive health care resource use, we also ran a scenario where the ethnicity variables were excluded from machine learning models in order to assess the trade-offs between potential stigma by ethnicity and model performance.

### Training/test datasets – in-sample or hold-out terms [18]

All machine learning and traditional regression models were trained using the same training dataset (80% of the sample) and tested on the same test dataset (20% of the sample) so that we could compare the predictive power of these models.

### Model evaluation

For evaluating predictive power with data that are unbalanced, we used sensitivity, specificity, positive predictive value (PPV, precision) and F1 score. Sensitivity is the true positive rate, measuring the power of the model to correctly identify high-cost people among people with CVD. Specificity is the true negative rate, measuring the ability to correctly identify people not belonging the high-cost group among people with CVD. F1 is a harmony score between sensitivity and PPV (i.e., equal to 2/(1/sensitivity + 1/PPV). We calculated these evaluation criteria for each model and used the F1 score as the main model evaluation criteria. AUC (Area under the Receiver Operating Characteristics curve) scores were also provided for model comparisons. Explanatory variables that contribute more to the predicted outcomes were identified using a Gini index, which provides a relative ranking of variable importance [46].

## Results

### Descriptive epidemiological and administrative data

As shown in Table 1, there were 1,889,000 New Zealanders aged 30–74 (the general population) with complete data on basic demographic information: age and sex. In 2014, 97,950 people had CVD (the study population, equals to 5.2% of the total population) and 149,000 people had diabetes (7.9%). As expected, compared to the general population, the study population was an older population (a mean age of 62.7 vs 49.7 years), had proportionately more males (66% vs 47%), had lower levels in paid employment (45% vs 71%), more ex-smokers (39% vs 25%), more people in low-income (59% vs 36%) and had a majority using CVD preventive medications. There were less than 0.5% of observations having missing ethnicity and deprivation data, and less than 10% missing smoking status and income data. All observations with missing data other than age and sex were included in the analyses and were implicitly treated as missing data.

### Descriptive health cost data among people with CVD

**Fig. 1:**
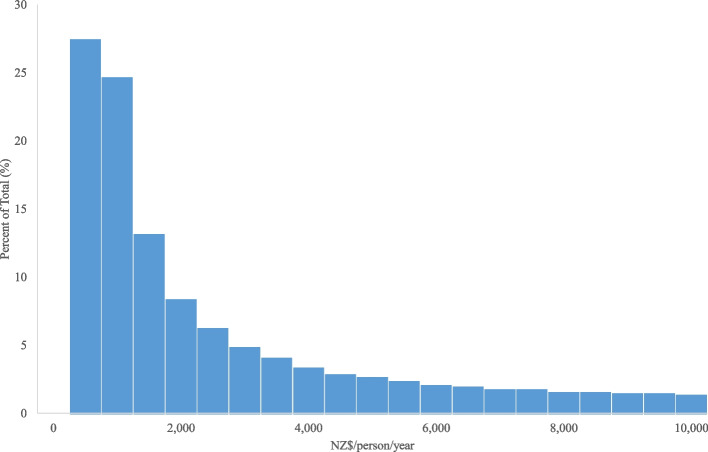
Health cost distribution (in the public health system, all health costs) among people with CVD in 2014 in New Zealand (NZ$/person/year)

Health cost distribution among people with CVD in New Zealand is very skewed to the right, with a mean cost of ~ NZ$4800/person/year (US$3320, €2510) and a median of NZ$1200/person/year (US$830, €627) (see Fig. 1, note that costs in this figure were trimmed at NZ$10,000/person/year (US$6920, €5230)).

### Main analysis

Table 2 presents predictive performance of identifying high-cost users among people with CVD for both traditional regression and machine learning models. The main traditional regression model (the model that included all well-established risk factors – model TRM1) achieved only a sensitivity of 4.9% (i.e., correctly identifying under 5% of high-cost people), a specificity of 99.2% (i.e., correctly identifying nearly all the non-high-cost people), a PPV of 61.2% (i.e., the proportion of the predicted high-cost users that was true high-cost users), a F1 score of 9.1%, and an AUC of 0.53. Detailed regression outputs were attached in the Appendix (Table S2).

**Table 2:** Predictive performance of identifying high-cost patients with CVD for traditional regression models versus machine learning models

Prediction models	Sensitivity^a^	Specificity^b^	Positive Predictive Value/ Precision^c^	Harmony score (sensitivity & positive predictive value), F1^d^	AUC^e^
**Traditional regression models**					
All conventional variables (TRM1)^f^	4.9%	99.2%	61.2%	9.1%	0.53
As per TRM1 but no ethnicity variables (TRM2)	4.9%	99.2%	62.5%	9.0%	0.53
As per TRM2 but no smoking variables (TRM3)	4.6%	99.2%	61.8%	8.6%	0.53
As per TRM3 but no chronic condition variables	0.0%	100%	Not calculable	Not calculable	0.53
**Machine learning models**^**g**^					
Random forest	37.8%	88.6%	45.2%	41.2%	0.70
KNN	38.0%	85.9%	40.1%	39.0%	0.45
L1-regularised logistic regression	78.9%	83.5%	22.1%	34.5%	0.62
Classification trees	19.5%	98.0%	71.2%	30.6%	0.73

Note: at 20% top high-cost users of all people with CVD

^a^correctly identified people with CVD who are high-cost = recall = true positive rates

^b^correctly identified people with CVD who are not high-cost = true negative rates

^c^true positives/false negatives = true positives among people who were predicted positives

^d^ = 2/(1/recall + 1/precision)

^e^Area under the Receiver Operating Characteristics curve

^f^age, sex, ethnicity, smoking status, other chronic conditions: diabetes, cancers and traumatic brain injuries

^g^all variables as per Table 1

All machine learning models outperformed the traditional regression models in sensitivity and F1 score, with the random forest model achieving a sensitivity of 37.8%, a specificity of 88.6%, a PPV of 45.2%, a F1 score of 41.2%, and an AUC of 0.70. Further predictive performance of the models are in the Appendix (Table S3). The L1-regularised logistic regression model achieved high sensitivity and specificity, but low PPV at 22.1%. The classification trees performed well at specificity and PPV (71.2%) but very low sensitivity (19.5%). The KNN model was just a bit below the random forest model at all evaluation measures. Of note is that the traditional regression model that included on all available independent variables as per these machine learning models did not provide stable estimates, i.e., there can be multiple model results in the same regression (due to sparing or identification issues).

**Fig. 2:**
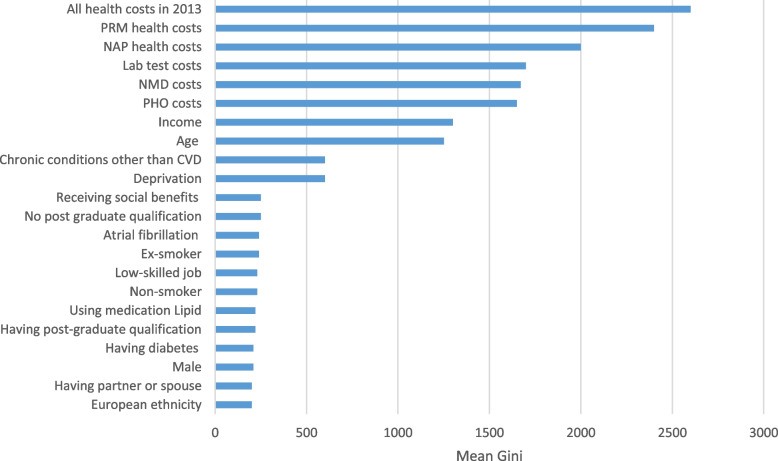
Important variables extracted from the random forest model. Notes: PRM = Pharmaceuticals; NAP = Non-admitted patients (i.e., outpatients and emergency department [ED] visits); Lab = Laboratory tests; NMD = Public hospitalisations (National minimum dataset); PHO = Primary health organisation (i.e., GP) enrolments. Gini index provides a relative ranking of variable importance [46]. Chronic conditions other than CVD: diabetes, cancers and traumatic brain injuries. Deprivation quintiles on a one to five scale with five being the most deprived

In terms of risk factors, excluding ethnicity (TRM2) and smoking status (TRM3) did not reduce the predictive performance of the traditional regression model. But long-term conditions were strong predictive factors for high-cost users among people with CVD (Table 2). For machine learning models, important variables extracted from the random forest model suggested that health costs in the previous year, be it laboratory tests or medication costs, played a critical role in predicting high-cost users (Fig. 2). These factors were then followed by income, age, deprivation, benefits received, and presence of other (non-CVD) long-term conditions. Ethnicity did not seem to play an important role in predicting high-cost users in this dataset. Of note is that it is not appropriate to interpret the associations of these variables with the outcome variable in this model (i.e., these variables had strong predictive power of the outcome in this particular random forest model, but do not necessary reflect a causal relationship).

### Sensitivity analysis with high-cost thresholds

Table 3 shows predictive performance for traditional regression models versus machine learning models in predicting high-cost users among people with CVD. When the threshold for high-cost users was expanded to 30% (i.e., the top 30% of patients with highest healthcare costs were considered as high-cost users), all model prediction improved in terms of sensitivity as expected but the traditional regression models improved more in relative terms (3.7 times (17.9%/4.9%) versus 2.4 times (46.1%/19.5%). However, the predictive performance of traditional models (the main model’s sensitivity 17.9%) was still far below that of machine learning models (the highest model sensitivity being 75.2%). At lower high-cost thresholds (i.e., the top 10% and 5% high-cost users), machine learning models also outperformed traditional regression models in terms of sensitivity and F1 score. At a 5% threshold, all traditional regression models performed no better than chance. The random forest model consistently performed better than other machine learning models at predicting less common outcomes (e.g., the top 5% high-cost users) based on the F1 scores.

**Table 3:** Sensitivity analysis for various high-cost user thresholds: predictive model performance

Prediction models	30% high-cost users prevalence		20% prevalence (the base case)		10% prevalence		5% prevalence	
	**Sensitivity**^**a**^	**F1**^**d**^	**Sensitivity**^**a**^	**F1**^**d**^	**Sensitivity**^**a**^	**F1**^**d**^	**Sensitivity**^**a**^	**F1**^**d**^
**Traditional regression models**								
All conventional variables (TRM1)^e^	17.9%	26.4%	4.9%	9.1%	*	*	*	*
As per TRM1 but no ethnicity variables (TRM2)	16.5%	25.8%	4.9%	9.0%	*	*	*	*
As per TRM2 but no smoking variables (TRM3)	16.3%	25.6%	4.6%	8.6%	*	*	*	*
**Machine learning models**^**f**^								
Random forest	45.2%	49.3%	37.8%	41.2%	29.9%	32.6%	25.6%	28.5%
KNN	45.7%	46.5%	38.0%	39.0%	29.2%	30.1%	25.2%	26.0%
L1-regularised logistic regression	75.2%	50.9%	78.9%	34.5%	72.5%	21.0%	76.2%	25.0%
Classification trees	46.1%	55.3%	19.5%	30.6%	11.4%	19.8%	10.9%	19.5%

Note: ^a^Results produced from the model were unstable due to a small number of CVD events in relation to the total observations

^a, b, c, d, e, f^: see Table 2

The results for the traditional regression model as per TRM3 but no chronic condition variables were not reported as this model had very poor predictive power

In term of risk factors, while previous healthcare costs, income, age, deprivation and benefits received were still dominant in predicting high-cost users, the lists of variables that contributed to the prediction outcome seem to slightly change with each high-cost user threshold beyond the top 15 variables (Appendix Figure S2-4). Importantly, when utilising these risk factors identified by machine learning models in the traditional regression model, it improved the predictive performance of this model significantly. For example, including previous healthcare costs increased predictive performance of a traditional regression model (model TRM1) to 17.9% for sensitivity, but this was still far below the best machine learning model with 75.2% sensitivity.

### Sensitivity analysis with inclusion of different sets of predictors

In contrast to the machine learning models, the traditional regression models could not deal with all variables available in this study due to identification issues (i.e., too many zeros or less common outcomes in the dataset). As expected, therefore machine learning techniques have the advantage of being able to deal with large datasets and a large number of independent variables more direct. When we ran machine learning models with the same limited set of independent variables as per the traditional regression models, the results suggested that machine learning models (in particular random forest and KNN) still outperformed the traditional regression models in terms of F1 score and AUC. For example, the KNN had an F1 score of 10% and AUC of 0.58 for KNN compared with an F1 score of 8% and AUC of 0.52 for the traditional model). See further details in the Appendix Table S4.

To deal with a potential ethnicity stigma (i.e., negative messages about a particular race [47]), in a sensitivity analysis, we excluded ethnicity variables from the full set of variables for machine learning models, and the results showed a very minor trade-off between model performance and potential ethnicity stigmatisation. The mean F1 scores and AUC values only slightly changed as a result of excluding the ethnicity variables from the models. For example, F1 score changed from 42 to 41%, and AUC reduced from 0.70 to 0.69 for the random forest model when the ethnicity variables were excluded. Further details are provided in Appendix Table S5.

## Discussion

In this study, we developed machine learning models to predict high-cost users among people with CVD using linked health and social administrative datasets at a national level. We found that machine learning models had substantially better predictive performance in terms of the key metric used (F1 scores) compared with the traditional cost regression models, for application in the same dataset as the training or learning occurred.

### Less common events

As expected, the machine learning models were more accurate than the traditional models in prediction of high-cost users in the cohort with highly skewed distribution of cost. That highlights the potential role of machine learning models in predicting uncommon healthcare events which could help to plan interventions for people who are most in need. Among machine learning models, we found that predictive performance of the random forest model consistently better at predicting less common outcomes. Furthermore, in line with the literature, even with the same limited set of independent variables as per the traditional regression models, our results suggested that some machine learning models still outperformed the traditional regression models [18].

### Risk factors for high-cost users among people with CVD

Our machine learning models were also able to identify important variables which are well-known risk factors of CVD, such as age, sex, chronic conditions, and behaviours such as smoking status. It must be emphasised that our models were designed for prediction and not for causal inference; hence, the important variables identified in this study should not be assumed to be causal for CVD high-cost users. Nevertheless, the results were able to suggest that high-cost utilisation seems to be persistent. This is in line with the results from a previous study [6] which showed that previous healthcare costs were strongly predictive of future healthcare costs.

As the set of important variables changed with the high-cost user thresholds, it is suggested that separate models might need to be built for different study populations so that variables of concerns can be identified and intervened. This is in line with the literature which suggests different high-cost user populations have different characteristics, for example, health and living conditions [1]. We provided a sensitivity analysis to the number of variables in Table 2 (different sets of predictors were used for the traditional regression models) and Table S4 (all machine learning models were built on the same limited number of predictors). Results in Table 2 suggested that the more variables were included, the better the model performance was observed as expected. One of the advantages of machine learning methods over traditional regression methods is that they can deal with a large number of variables; however, even with a limited set of variables as per Table S4, the top machine learning models KNN and random forest still outperformed the traditional regression model in the core metrics F1 score and AUC. As discussed, the aim of our study was to focus on prediction tasks, therefore which parameters drive the superior performance of machine learning methods would be an avenue for future research. In our views, it could be the large number of variables, interactions among variables or the functional forms that drive the superior performance. This is known as the interpretability of the models or explainable machine learning, which has been actively researched in the literature [48–50].

In addition to well-established risk factors for high cost users, relatively new variables such as benefits received, deprivation, CVD medications, owning a house, and post-graduate qualifications were identified in machine learning models as important factors. This suggests that the use of machine learning models could contribute to the identification of new risk factors for specific diseases (i.e., further investigating these new variables using a causal inference method) [24, 51]. Furthermore, the more variables were included in regression models, the better performance of the models was (but this can also lead to overfitting, i.e., poor performance on a new dataset). The advantage of using machine learning is that it can help to identify new variables quickly in massive datasets without doing step-wise regression or having to specify functional forms in high-dimensional settings as in traditional models [20, 52–54]. Of note is that including a large number of independent variables into a traditional regression model may not work due to sparing or identification issues (i.e., too many observations with zero values).

We found that ethnicity did not seem to play an important role in predicting high-cost users in our study which used linked administrative dataset, as opposed to disease risk prediction models in New Zealand [44, 55]. This could be due to ethnic differences being largely mediated through the inclusion of the measures of deprivation, income, educational level and social security benefits received [56, 57]. Of note is that the aim of our sensitivity analysis which removed ethnicity variables was to provide evidence and promote discussion with regard to trade-offs between model performance and stigmatisation by ethnicity. Using ethnicity variables as a proxy covariate in health economic regression models might not be an optimal choice to address health inequities by ethnicity [58].

### Study strengths and limitations

We were able to use the Stats NZ IDI which is a comprehensive national-level database of linked administrative social and health datasets. This data infrastructure captures not only the health data but also extensive socio-economic and behavioural data. To our knowledge, this is the first study to use machine learning for prediction of CVD high-cost user using a national-level dataset with a rich set of health and social variables. We were also able to compare the machine learning models with the traditional regression models with various high-cost user thresholds. Since computers are now involved in many health system and economic transactions, big data is becoming increasingly available. Data manipulation tools and techniques developed for small datasets will become increasingly insufficient to address important research questions [20].

However, it should be emphasised that we only employed traditional regression models as a comparison to machine learning models. Econometrics and statistical methods have been developed over many decades; these techniques are no doubt still useful for answering many health economic questions, such as estimating parameters or size effects. Machine learning models as built in this study can, however, be used as a new tool to analyse large datasets or as a complement to well-developed econometric and statistical techniques.

Our sensitivity analysis suggested that there was a very minor trade-off between model performance and potential ethnicity stigma by excluding ethnicity variables from the full set of variables for machine learning models. This finding may be of interest internationally as ethnicity information is not routinely collected in some other high-income countries such as Germany and Sweden. In these countries, proxy ethnicity data are collected in the form of migration such as German and foreigner (with and without migration background, and with and without migration experience); and foreign origin (persons born outside of Sweden or persons born in Sweden who’s both parents were born outside of Sweden) as opposed to Swedish origin [59].

We did not test all available machine learning techniques such as those with different function classes and feature representations (i.e., variable coding or classification) as there are no definitive and universal guidelines on the best practices [18]. Furthermore, the scope of our study was to explore the efficiency of this new econometrics tool (i.e., machine learning methods). But given our dataset (with over 100,0000 observations) being much larger than those commonly used in the literature, [60] it probably has more generalisation capacity. Some techniques such as cross-validation did not seem to improve the reliability of the prediction in large datasets (as checked in this study), holding everything else constant [35, 61, 62].

Our traditional regression model (TRM3, Table 2) with only sex, age and chronic conditions as predictors can be used as a proxy for the baseline model, and hence its metrics can be regarded as baseline scores. Studies that have analysed health costs or have modelled health economic outcomes have commonly utilised these variables [1, 3, 63]. As a result, we can also conclude that machine learning methods appear better than the baseline model in our analysis.

We provided different metrics as this is an interdisciplinary research area and we believe that researchers from different disciplines may be familiar with different metrics. We chose F1 score as our main metric as it fits with our prediction task and the characters of the data. One may choose to use other metrics such as F-beta score or Matthews correlation coefficient. F1 score is similar to F-beta score in the sense that it also takes into account both sensitivity and precision, but it can be considered as a special case of F-beta when beta equals 1 that is an equal weight. We provided sensitivity and precision in our results so readers who choose to give more weight to either of these can calculate the corresponding F-beta. Matthews correlation coefficient takes into account true and false positives and negatives, and is useful for unbalanced classes. But, in this case, as negative is the majority class and we were more interested in the positive class, F1 is good enough (as it places emphasis on the positive class), and F1 weights sensitivity and precision in a more interpretable way than Matthews correlation does.

CVD costs can vary significantly with sub-conditions and stages of diseases; however, we did not distinguish coronary heart disease costs from stroke costs, which are potentially more expensive.

While we found that machine learning methods performed better than traditional regression models using administrative health and socio-economic data, there is evidence that logistic regression models perform as well as machine learning models in clinical prediction models [64]. However, a CVD risk prediction model using NZ data found that deep learning extensions of survival analysis models were more accurate than traditional Cox proportional hazards models [65].

Both machine learning and traditional regression models in this study were built for prediction tasks so we did not assess omitted variable bias, heteroscedasticity or endogeneity issues as normally checked in causal inference models.

### Potential implications for health system planners and managers

This work suggests that by improving prediction of high-cost users, machine learning might help with health services planning to better match future demand for services. Furthermore, as administrative linked data get better and involve larger datasets, the machine learning approach can potentially help to ease manual tasks by health researchers and planners (such as selecting variables, checking variable correlations, and specifying functional forms for regression equations) in health costs prediction.

In addition, this work also suggests that machine learning may allow for improved risk factor identification. This can lead to better targeted preventive interventions directed at groups at highest risk (e.g., better use of preventive medicines, smoking cessation support, and improved management of comorbidities such as diabetes). This could then enhance the cost-effectiveness of service delivery by achieving health gain in those most in need, and also contribute to reducing health inequalities. The focus on improving health of potential high-cost users may also ultimately reduce the need for avoidable health expenditure.

## Conclusions

This study provides additional evidence that machine learning can be used as a tool in health economics together with econometric methods for both prediction and identification of new risk factors. Our findings suggest that machine learning, in conjunction with access to big data, can better predict people with CVD who will become high-cost users of the health system compared to traditional approaches. By improving prediction of high-cost users, the use of machine learning may help with health services planning and preventive measures to improve population health while potentially saving healthcare costs.

## Supplementary Information

**Additional file 1: Appendix.**

## Data Availability

Access to the anonymised data used in this study was provided by Stats NZ under the security and confidentiality provisions of the Statistics Act 1975. Only people authorised by the Statistics Act 1975 are allowed to see data about a particular person, household, business, or organisation, and the results in this paper have been confidentialised to protect these groups from identification and to keep their data safe.

## Abbreviations

AUC: Area under the Receiver Operating Characteristics curve
CVD: Cardiovascular diseases
IDI: Integrated Data Infrastructure
KNN: K-nearest neighbourhood
PPV: Positive predictive value
TRM: Traditional regression model

## Code availability

Code available upon request but note that data for running this code are only available in a strict confidential environment managed by Stats NZ (New Zealand).
