# Supplementary material for: Predicting high health-cost users among people with cardiovascular disease using machine learning and nationwide linked social administrative datasets
Source: Health Econ Rev. 2023 Feb 4;13:9. doi: 10.1186/s13561-023-00422-1 (PMC9898915; doi:10.1186/s13561-023-00422-1)
Supplement: Supplementary file 1 — Additional file 1: Appendix. [file 13561_2023_422_MOESM1_ESM.docx]

# Appendix

**Table S1. List of independent variables (i.e., covariates or features) used in machine learning models**

| **Variable names** | **Definitions and/or ICD-10 codes** |
| --- | --- |
| Male |  |
| Female |  |
| Age in year |  |
| Ethnicity Māori (Indigenous New Zealanders) |  |
| Ethnicity non Māori |  |
| Ethnicity European |  |
| Ethnicity Pacific peoples |  |
| Ethnicity Asian |  |
| Income |  |
| High income | High-income (>NZ$70,000/year) |
| Medium income | Medium-income (>=NZ$30,000/year & <=NZ$70,000/year (US$48,400, €36,600)) |
| Low income | Low-income (<NZ$30,000/year (US$20,750, €15690)) |
| On benefits |  |
| Having an offence record |  |
| NZ Deprivation low decile |  |
| NZ Deprivation mid decile |  |
| NZ Deprivation high decile |  |
| NZ Deprivation quintile | Value between 1 and 5 |
| Birth country Oceania |  |
| Birth country North West Europe |  |
| Birth country South East Europe |  |
| Birth country North Africa and Middle East |  |
| Birth country South East Asia |  |
| Birth country North East Asia |  |
| Birth country Southern Central Asia |  |
| Birth country Americas |  |
| Birth country Sub Saharan |  |
| Current smoker |  |
| Ex-smoker |  |
| Non smoker |  |
| High skilled job |  |
| Low skilled job |  |
| Medium skilled job |  |
| Speaking another language not English not Te Reo (Indigenous language) |  |
| Official language Te Reo |  |
| Official language English |  |
| Having a spouse/partner |  |
| Single |  |
| Having stressful life events |  |
| In paid employment |  |
| No postgraduate qualification |  |
| Having postgraduate qualification |  |
| Owning a house |  |
| Rent usual residence |  |
| Use of CVD preventive medications in 2013 (at least 2 prescriptions): Any anti-hypertensive medication | 'Calcium Channel Blockers', 'Diuretics', 'Agents Affecting the Renin-Angiotensin System', 'Beta Adrenoceptor Blockers', ' Beta Adrenoceptor Blockers with Diuretics' |
| Use of CVD preventive medications in 2013 (at least 2 prescriptions): Any lipid-lowering medication | 'Lipid Modifying Agents' |
| Use of CVD preventive medications in 2013 (at least 2 prescriptions): Any prescribed anti-thrombotic agent | 'Antithrombotic Agents' |
| Use of CVD preventive medications in 2013 (at least 2 prescriptions): Any diabetes medication |  |
| Having Atrial fibrillation | I48 |
| Having Hypertension | I10 |
| Having CVD prior 2014 | CHD Definition: A healthcare user is counted as having CHD if they meet any one of the following conditions: 1. National Minimum Dataset (NMDS) diagnosis codes: ICD-10-AM: I20-I25  Stroke Definition: where the healthcare user had a publicly funded hospital discharge in a primary diagnosis of I60-164 (ICD-10-AM-II). Further information see data dictionary[66]. |
| Having diabetes prior 2014 | E10–E14 (further information see data dictionary[66]) |
| Having other chronic prior 2014 | Cancers, traumatic brain injuries, and gout (further information see data dictionary[66]) |

# Table S2. Regression results for a traditional regression model (TRM1)

> model <- glm(y_train~ Age_in_year + male + current_smoker+ex_smoker

+non_smoker+Ethnicity_Maori + Ethnicity_European

+Ethnicity_Pacific + Ethnicity_Asian + Have_diabetes_prior_2014 + Have_Atrial_fibrillation

+ Have_Hypertension +Have_other_chronic_prior_2014, data=x_train, family=binomial(link=logit))

Deviance Residuals:

Min 1Q Median 3Q Max

-1.6332 -0.6841 -0.5558 -0.4501 2.3925

Coefficients: (1 not defined because of singularities)

Estimate Std. Error z value Pr(>|z|)

(Intercept) -2.893020 0.095469 -30.303 < 2e-16 ***

Age_in_year 0.017648 0.001203 14.671 < 2e-16 ***

male -0.190400 0.019608 -9.710 < 2e-16 ***

current_smoker 0.059694 0.047892 1.246 0.2126

ex_smoker -0.084145 0.043662 -1.927 0.0540 .

non_smoker -0.207757 0.043625 -4.762 1.91e-06 ***

Ethnicity_Maori 0.068278 0.039125 1.745 0.0810 .

Ethnicity_€opean -0.041472 0.039375 -1.053 0.2922

Ethnicity_Pacific 0.131307 0.058450 2.247 0.0247 *

Ethnicity_Asian -0.111918 0.057316 -1.953 0.0509 .

Have_diabetes_prior 0.634958 0.019786 32.091 < 2e-16 ***

Have_Atrial_fib. 0.811906 0.025163 32.265 < 2e-16 ***

Have_Hypertension 0.501165 0.089582 5.594 2.21e-08 ***

Have_other_chronic 0.364352 0.011434 31.865 < 2e-16 ***

---

Signif. codes: 0 ‘***’ 0.001 ‘**’ 0.01 ‘*’ 0.05 ‘.’ 0.1 ‘ ’ 1

(Dispersion parameter for binomial family taken to be 1)

Null deviance: 78450 on 78360 degrees of freedom [Of note is that this number equals to the size of the training dataset, that is 80% of the total study population (N=97,950 observations)]

Residual deviance: 74100 on 78360 degrees of freedom

AIC: 74130

Number of Fisher Scoring iterations: 4

# Table S3. Prediction results for both traditional regression and machine learning models with sensitivity analysis

| **Prediction models** | |  | **Traditional regression models** | | **Machine learning models^f^** | | | |
| --- | --- | --- | --- | --- | --- | --- | --- | --- |
|  |  | All conventional variables (TRM1)^e^ | As per TRM1 but no ethnicity variables (TRM2) | As per TRM2 but no smoking variables (TRM3) | L1-regularised logistic regression | Classification trees | KNN | Random forest |
| **30% high health cost users prevalence** | Sensitivity^a^ | 17.9% | 16.5% | 16.3% | 75.2% | 46.1% | 45.7% | 45.2% |
|  | Specificity^b^ | 95.1% | 95.1% | 95.4% | 78.4% | 91.2% | 78.6% | 83.9% |
|  | PPV/ Precision^c^ | 59.8% | 59.3% | 60.4% | 38.5% | 69.0% | 47.5% | 54.2% |
|  | F1^d^ | 26.4% | 25.8% | 25.6% | 50.9% | 55.3% | 46.5% | 49.3% |
|  | AUC | 0.56 | 0.56 | 0.56 | 0.66 | 0.73 | 0.48 | 0.70 |
| **20% prevalence (the base case)** | Sensitivity^a^ | 4.9% | ­­4.9% | 4.6% | 78.9% | 19.5% | 38.0% | 37.8% |
|  | Specificity^b^ | 99.2% | 99.2% | 99.2% | 83.5% | 98.0% | 85.9% | 88.6% |
|  | PPV/ Precision^c^ | 61.2% | 62.5% | 61.8% | 22.1% | 71.2% | 40.1% | 45.2% |
|  | F1^d^ | 9.1% | 9.0% | 8.6% | 34.5% | 30.6% | 39.0% | 41.2% |
|  | AUC | 0.53 | 0.53 | 0.53 | 0.62 | 0.73 | 0.45 | 0.70 |
| **10% prevalence** | Sensitivity^a^ | * | * | * | 72.5% | 11.4% | 29.2% | 29.9% |
|  | Specificity^b^ | * | * | * | 91.2% | 99.7% | 92.9% | 94.2% |
|  | PPV/ Precision^c^ | * | * | * | 12.3% | 78.5% | 31.1% | 35.8% |
|  | F1^d^ | * | * | * | 21.0% | 19.8% | 30.1% | 32.6% |
|  | AUC | 0.50 | 0.50 | 0.50 | 0.57 | 0.62 | 0.45 | 0.67 |
| **5% prevalence** | Sensitivity^a^ | * | * | * | 76.2% | 10.9% | 25.2% | 25.6% |
|  | Specificity^b^ | * | * | * | 95.8% | 100.0% | 96.5% | 97.2% |
|  | PPV/ Precision^c^ | * | * | * | 15.0% | 92.1% | 26.9% | 32.2% |
|  | F1^d^ | * | * | * | 25.0% | 19.5% | 26.0% | 28.5% |
|  | AUC | 0.50 | 0.50 | 0.50 | 0.58 | 0.58 | 0.43 | 0.67 |

*Note: * Results produced from the model were unstable due to a small number of CVD events in relation to the total observations.*

*^a, b, c, d, e, f^: see Table 2.*

*^The results for the traditional regression model as per TRM3 but no chronic condition variables were not reported as this model had very poor predictive power.^*

# Table S4. Prediction results when a limited set of variables were used for both traditional regression and machine learning models

| **Evaluation criteria** | **Traditional regression models** | **Machine learning models^f^** | | | |
| --- | --- | --- | --- | --- | --- |
|  | All conventional variables (TRM1)^e^ | L1-regularised logistic regression | Classification trees | KNN | Random forest |
| Sensitivity^a^ | 55.3% | 0.1% | Not calculable | 50.9% | 52.1% |
| Specificity^b^ | 80.9% | 100.0% | 80.3% | 82.2% | 81.0% |
| PPV/ Precision^c^ | 4.5% | 100.0% | 0.0% | 15.2% | 5.6% |
| F1^d^ | 8.2% | 0.1% | Not calculable | 23.4% | 10.1% |
| AUC | 0.52 | 0.50 | 0.50 | 0.58 | 0.54 |

*Note: ^a, b, c, d, e, f^: see Table 2.*

# Table S5. Prediction results for both traditional regression and machine learning models with ethnicity variables excluded

| **Evaluation criteria** | **Traditional regression models** | **Machine learning models^f^** | | | |
| --- | --- | --- | --- | --- | --- |
|  | All conventional variables (TRM1)^e^ | L1-regularised logistic regression | Classification trees | KNN | Random forest |
| Sensitivity^a^ | 56.7% | 21.4% | 57.7% | 40.2% | 45.0% |
| Specificity^b^ | 80.9% | 98.2% | 84.8% | 84.9% | 85.3% |
| PPV/ Precision^c^ | 4.7% | 74.4% | 31.4% | 37.3% | 37.6% |
| F1^d^ | 8.7% | 33.3% | 40.6% | 38.7% | 41.0% |
| AUC | 0.52 | 0.60 | 0.70 | 0.46 | 0.69 |

*Note: ^a, b, c, d, e, f^: see Table 2.*


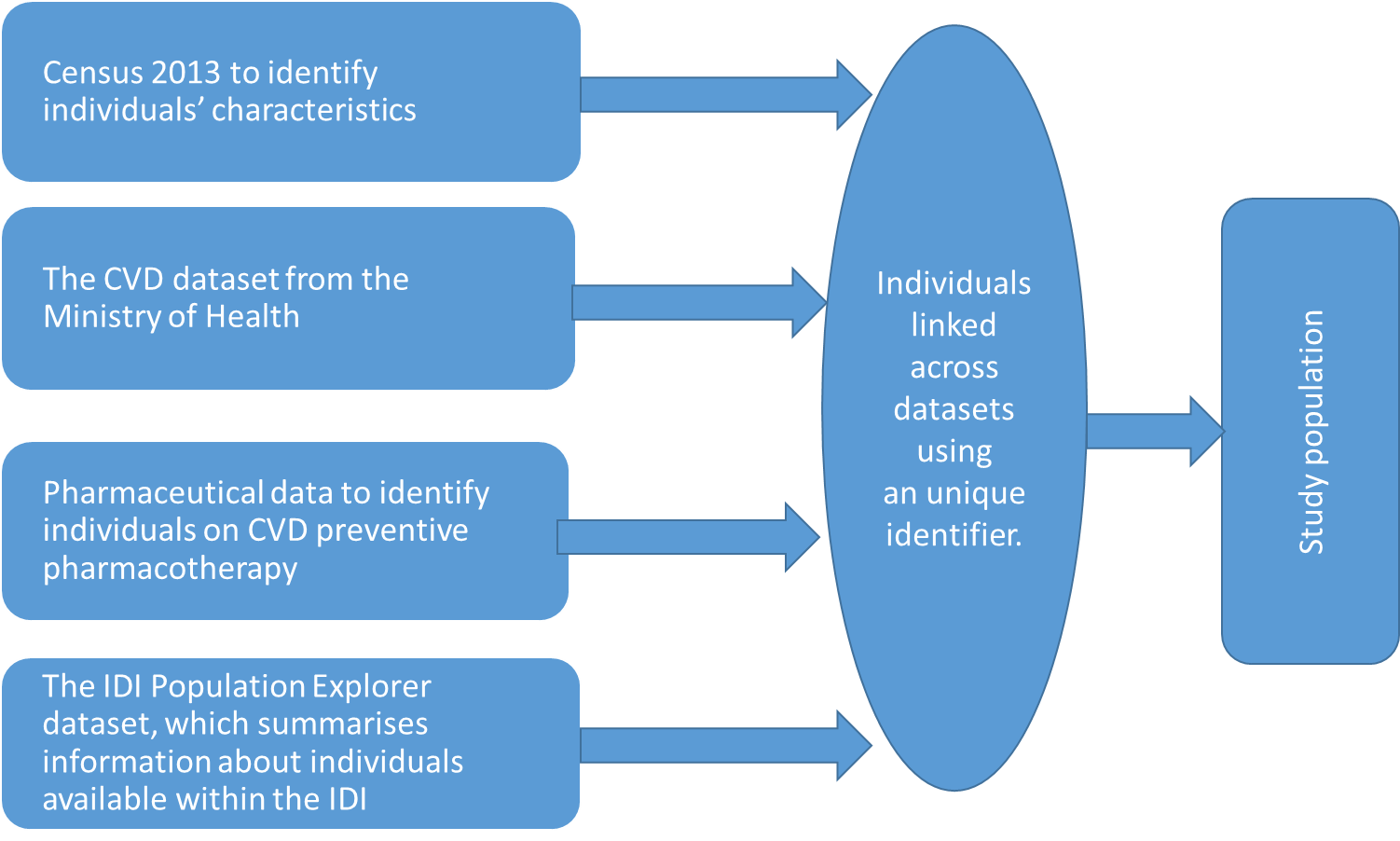


**Figure S1. Datasets and data linkage in the IDI to create study population and independent variables**

# Figure S2. Important variables from a sensitivity analysis of 30% high-cost user prevalence

*Notes*: PRM=Pharmaceuticals; NAP=Non-admitted patients (i.e., outpatients and ED visits); Lab=Laboratory tests; NMD=Public hospitalisations (National minimum dataset); PHO=Primary health organisation (i.e., GP) enrolments. Chronic conditions other than CVD: diabetes, cancers and traumatic brain injuries. Deprivation quintiles on a one to five scale with five being the most deprived.

# Figure S3. Important variables from a sensitivity analysis of 10% high-cost user

*Notes*: PRM=Pharmaceuticals; NAP=Non-admitted patients (i.e., outpatients and ED visits); Lab=Laboratory tests; NMD=Public hospitalisations (National minimum dataset); PHO=Primary health organisation (i.e., GP) enrolments. Chronic conditions other than CVD: diabetes, cancers and traumatic brain injuries. Deprivation quintiles on a one to five scale with five being the most deprived.

# Figure S4. Important variables from a sensitivity analysis of 5% high-cost user prevalence

*Notes*: PRM=Pharmaceuticals; NAP=Non-admitted patients (i.e., outpatients and ED visits); Lab=Laboratory tests; NMD=Public hospitalisations (National minimum dataset); PHO=Primary health organisation (i.e., GP) enrolments. Chronic conditions other than CVD: diabetes, cancers and traumatic brain injuries. Deprivation quintiles on a one to five scale with five being the most deprived.

**Reference**

66. Stats NZ. IDI MOH Chronic Condition/Significant Health Event Cohort data, URL: <https://datainfoplus.stats.govt.nz/Item/nz.govt.stats/ac775e86-9f66-486a-adb9-64b0f512c54c>. 2015 [Accessed 7 May 2022].
